# Impact of behavioral and psychological symptoms of Alzheimer’s disease on caregiver outcomes

**DOI:** 10.1038/s41598-022-18470-8

**Published:** 2022-08-19

**Authors:** Kanokporn Pinyopornpanish, Atiwat Soontornpun, Tinakon Wongpakaran, Nahathai Wongpakaran, Surat Tanprawate, Kanokwan Pinyopornpanish, Angkana Nadsasarn, Manee Pinyopornpanish

**Affiliations:** 1 Department of Family Medicine, Faculty of Medicine, Chiang Mai University, Chiang Mai, Thailand; 2 Division of Neurology, Department of Internal Medicine, Faculty of Medicine, Chiang Mai University, 110 Inthawarorot Rd., Sriphum, Muang, Chiang Mai, 50200 Thailand; 3 Northern Neuroscience Center, Faculty of Medicine, Chiang Mai University, Chiang Mai, Thailand; 4 Department of Psychiatry, Faculty of Medicine, Chiang Mai University, Chiang Mai, Thailand

**Keywords:** Alzheimer's disease, Geriatrics

## Abstract

This study was to determine the prevalence of behavioral and psychological symptoms of dementia (BPSD) and its association with dementia severity and to explore the association between specific BPSD and caregiver stress, burden, and depression. A cross-sectional study involving the interviewing of the primary caregivers of patients with Alzheimer’s disease (AD) was conducted. Multivariable analysis was used to analyze the associations between specific symptoms of BPSD and caregiver outcomes. A total of 102 AD patients (age 79.4 ± 7.9 years, 70.6% female) and their caregivers were included. Nearly 46% had moderate-to-severe AD. Nearly all patients (99.0%) had at least one BPSD. Apathy was among the most common symptoms (74.5%), and hallucination was the only symptom associated with severity of AD (*p* = 0.017). After adjustment, agitation was associated with Patient Health Questionnaire-9 (PHQ-9) and Zarit Burden Interview (ZBI-22) (*p* = 0.021 and 0.007, respectively); sleep disorders were associated with only PHQ-9 (*p* = 0.049). In conclusion, the BPSD, especially agitation and sleep disorders, can give rise to difficulties for both patients and their caregivers. The prevalence of BPSD is high (99.0%), and the symptoms can start early. Routine screening of BPSD in all AD patients is advocated.

## Introduction

Alzheimer’s disease (AD) is associated with progressive, irreversible cognitive decline and is the most common cause of dementia, being the cause of 60–70% of dementia cases either by itself or in combination with other disorders^1^. The prevalence is around 24 million and the predicted trend is to double every 20 years till 2040^2^. Patients with AD mostly suffer from a decrease in cognitive function including deterioration in memory, difficulty in word-finding, impaired reasoning, and an increase in visuospatial problems. In mild AD, patients are able to maintain most of their activities of daily living but late in the course of the illness, they need more help to complete many activities associated with daily life and the majority ending up with total dependency^3,4^.

In addition to cognitive problems, patients with AD can display behavioral and psychological symptoms of dementia (BPSD). BPSD are commonly presented in up to 90% of patients with dementia. Whilst there are differences in presentation in different types of dementia with different degree of severity, patients with AD also experience these BPSD including aggression, psychotic symptoms, and other mood disorders and behavioral problems^5,6^.

In 2015, the estimated number of people with dementia was 600,000 in Thailand^7^. Informal care plays a significant role in caring for dementia patients in Asian countries including Thailand^8^. Caregiving and caregiver burden were different across different Asian countries^9^. A study using Caregiver Burden Inventory questionnaire found high burden for caring Thai dementia patients particularly those caring for dependent patients^10^. Poor quality of life and high levels of anxiety and depression among Thai dementia caregivers have been reported^11^. Evidence also suggests that, together with cognitive decline, BPSD could affect both patient outcomes and the lives of their caregivers^12^. Caregiver distress increased with increasing number and magnitude of BPSD^13,14^. Moreover, reduction in caregiver quality of life is associated with the presence of BPSD^15^. BPSD are associated with higher mortality rate and are a leading cause of institutionalization^16,17^.

A prior study demonstrated that the overall Neuropsychiatric Inventory Questionnaires (NPI-Q) score, an instrument used for assessing BPSD, is associated with caregiver burden through caregiver stress and depression^18^. However, inconsistent results about the association between specific symptoms of BPSD and psychosocial outcomes in caregivers have been found among studies^19,20^. As each patient could be affected by different domains of BPSD, knowledge concerning which domain affects the caregiver the most would be useful for clinical practice. This information would encourage the physicians or healthcare team to pay more attention to screening and treating BPSD. Few studies have characterized BPSD in accordance with dementia etiology^21^, therefore, the aim of this study is to determine BPSD prevalence in AD and its association with dementia severity. We also aim to explore the association between specific BPSD and caregiver stress, burden, and depression.

## Results

A total of 102 AD patients (age 79.37 ± 7.90 years, 70.59% female) and their caregivers were included. Forty-six (45.10%) had moderate-to-severe AD. Demographics of patients with AD and their caregivers including all assessment results of patients [Functional Assessment Staging Tool (FAST)^22^, Barthel index^23^, and NPI-Q^24^] and caregiver outcomes [Perceived Stress Scale (PSS)^25^, Patient Health Questionnaire-9 (PHQ-9)^26^, and Zarit Burden Interview (ZBI-22)^27^] are shown in Table 1. Mean duration of disease was 2.53 years (SD 3.14). One hundred and one patients (99.02%) exhibited at least one BPSD. Two patients (1.96%) had all twelve symptoms. The number of BPSD and severity of BPSD stratified by severity of AD are shown in Tables 2 and 3.

**Table 1:** Demographics and clinical characteristics of patients with AD and their caregivers.

Patients’ characteristics	n = 102
**Female gender, n (%)**	72 (70.59)
**Age, mean ± SD**	79.37 ± 7.90
**Marital status, n (%)**	
Married	44 (43.14)
Widow/widower	51 (50.00)
Single	5 (4.90)
Divorced/separated	2 (1.96)
**Duration of disease (years), mean ± SD**	2.53 ± 3.14
**AD type, n (%)**	
AD	63 (61.76)
AD + CVD	39 (38.24)
**Severity of disease by FAST, n (%)**	
Mild AD	56 (54.90)
Moderate-to-severe AD	46 (45.10)
**ADL, mean ± SD**	15.36 ± 6.47
**BPSD**	
Number of BPSD, mean ± SD	5.79 ± 2.66
NPI-Q severity score, mean ± SD	11.19 ± 6.22

Caregivers’ characteristics	n = 102
**Female gender, n (%)**	79 (77.45)
**Age, mean ± SD**	55.02 ± 12.89
**Relationship with patient, n (%)**	
Spouse	21 (20.6)
Parent	1 (1.0)
Child	70 (68.6)
Other relatives	5 (4.9)
Hired professional caregiver	5 (4.9)
**Years of education, mean ± SD**	13.78 ± 4.57
**PSS, mean ± SD**	14.88 ± 7.10
**PHQ-9, mean ± SD**	4.11 ± 3.96
**ZBI-22, mean ± SD**	18.44 ± 14.27

*AD* Alzheimer’s disease, *ADL* activity of daily living, *BPSD* behavioral and psychological symptoms of dementia, *CVD* cerebrovascular disease, *FAST* functional assessment staging test, *NPI-Q* Neuropsychiatric inventory questionnaires, *PSS* perceived stress scale, *PHQ-9* patient health questionnaire-9, *SD* standard deviation, *ZBI-22* 22-item Zarit Burden Interview.

**Table 2:** Numbers of BPSD based on 12 domains in NPI-Q.

Number of BPSD	Total n = 102	%	Mild AD n = 56	%	Moderate-to-Severe AD n = 46	%
0	1	0.98	1	1.79	0	0
1	3	2.94	0	0	3	6.52
2	6	5.88	3	5.36	3	6.52
3	10	9.80	5	8.93	5	10.87
4	17	16.67	12	21.43	5	10.87
5	13	12.74	8	14.29	5	10.87
6	15	14.70	10	17.86	5	10.87
7	7	6.86	2	3.57	5	10.87
8	11	10.78	7	12.50	4	8.70
9	10	9.80	3	5.36	7	15.22
10	5	4.90	2	3.57	3	6.52
11	2	1.96	2	3.57	0	0
12	2	1.96	1	1.79	1	2.17
At least one BPSD	101	99.02	55	98.21	46	100

*AD* Alzheimer’s disease, *BPSD* behavioral and psychological symptoms of dementia, *NPI-Q* neuropsychiatric inventory questionnaires.

**Table 3:** Prevalence of BPSD and severity of AD by FAST.

BPSD	Severity of BPSD	Total n = 102 (%)	Mild AD n = 56 (%)	Moderate-to-Severe AD n = 46 (%)	*p*-value
Delusions	Mild	10 (9.80)	4 (7.14)	6 (13.04)	0.166^b^
n = 40 (39.2%)	Moderate	17 (16.67)	6 (10.71)	11 (23.91)	
	Severe	13 (12.75)	9 (16.07)	4 (8.70)	
Hallucinations	Mild	13 (12.75)	7 (12.50)	6 (13.04)	0.017^b^
n = 34 (33.3%)	Mod	16 (15.69)	6 (10.71)	10 (21.74)	
	Severe	5 (4.90)	0 (0)	5 (10.87)	
Agitation	Mild	28 (27.45)	17 (30.36)	11 (23.91)	0.485^b^
n = 56 (54.9%)	Mod	18 (17.65)	7 (12.50)	11 (23.91)	
	Severe	10 (9.80)	5 (8.93)	5 (10.87)	
Dysphoria	Mild	27 (26.47)	18 (32.14)	9 (19.57)	0.514^b^
n = 42 (41.2%)	Mod	10 (9.80)	5 (8.93)	5 (10.87)	
	Severe	5 (4.90)	2 (3.57)	3 (6.52)	
Anxiety	Mild	17 (16.67)	8 (14.29)	9 (19.57)	0.873^a^
n = 47 (46.1%)	Mod	19 (18.63)	10 (17.86)	9 (19.57)	
	Severe	11 (10.78)	6 (10.71)	5 (10.87)	
Euphoria	Mild	9 (8.82)	4 (7.14)	5 (10.87)	0.411^b^
n = 16 (15.7%)	Mod	2 (1.96)	2 (3.57)	0 (0)	
	Severe	5 (4.90)	4 (7.14)	1 (2.17)	
Apathy	Mild	23 (22.55)	15 (26.79)	8 (17.39)	0.296^a^
n = 76 (74.5%)	Mod	24 (23.53)	15 (26.79)	9 (19.57)	
	Severe	29 (28.43)	12 (21.43)	17 (36.96)	
Disinhibition	Mild	16 (15.69)	10 (17.86)	6 (13.04)	0.860^b^
n = 38 (37.3%)	Mod	14 (13.73)	7 (12.50)	7 (15.22)	
	Severe	8 (7.84)	5 (8.93)	3 (6.52)	
Irritability	Mild	21 (20.59)	11 (19.64)	10 (21.74)	0.918^a^
n = 71 (69.6%)	Mod	30 (29.41)	18 (32.14)	12 (26.09)	
	Severe	20 (19.21)	11 (19.64)	9 (19.57)	
Aberrant motor behavior	Mild	18 (17.65)	11 (19.64)	7 (15.22)	0.947^a^
n = 52 (51.0%)	Mod	13 (12.75)	7 (12.50)	6 (13.04)	
	Severe	20 (19.61)	11 (19.64)	9 (19.57)	
Sleep disorders	Mild	14 (13.73)	7 (12.50)	7 (15.22)	0.363^a^
n = 68 (66.7%)	Mod	30 (29.41)	13 (23.21)	17 (36.96)	
	Severe	24 (23.53)	11 (19.64)	10 (21.74)	
Appetite/eating behavior	Mild	11 (10.78)	6 (10.71)	5 (10.87)	0.374^a^
n = 51 (50.0%)	Mod	22 (21.57)	15 (26.79)	7 (15.22)	
	Severe	18 (17.65)	11 (19.64)	7 (15.22)	
Total NPI-Q mean ± SD		11.19 ± 6.22	10.89 ± 5.85	11.54 ± 6.69	0.602^c^

*AD* Alzheimer’s disease, *BPSD* behavioral and psychological symptoms of dementia, *FAST* functional assessment staging test, *NPI-Q* neuropsychiatric inventory questionnaires.

^a^Chi-square test.

^b^Fisher’s exact test.

^c^student t-test.

Five most commonly reported BPSD were apathy (74.5%), irritability (69.6%), sleep disorders (66.7%), agitation (54.9%), and aberrant motor behavior (51.0%) (Table 3). The only symptom associated with the severity of AD was hallucination (*p* = 0.017). There was no association between severity of AD and other BPSD including delusion, agitation, dysphoria, anxiety, euphoria, apathy, disinhibition, irritability, abberent motor behavior, sleep disorder, and eating behavior.

The associations between individual BPSD and caregiver outcomes are shown in Table 4. Agitation (r 0.339, *p* < 0.001), apathy (r 0.256, *p* 0.009), disinhibition (r 0.231, *p* 0.019), irritability (r 0.253, *p* 0.010), and sleep disorders (r 0.222, p 0.025) were associated with greater PSS score. Agitation (r 0.333, *p* < 0.001), dysphoria (r 0.248, *p* 0.012), and sleep disorders (r 0.290, *p* 0.003) were associated with greater PHQ-9 score. Associations between greater ZBI-22 score with hallucination (r 0.220, *p* 0.027), agitation (r 0.460, *p* < 0.001), apathy (r 0.223, *p* 0.025), disinhibition (r 0.285, *p* 0.004), irritability (r 0.256, *p* 0.009), and sleep disorders (r 0.284, *p* 0.004) were found.

**Table 4:** Correlation between variables.

	1	2	3	4	5	6	7	8	9	10	11	12	13	14	15	16	17	18
1.Severity of AD	–																	
2.NPI-Q score	0.052	–																
3.Delusion	0.045	0.483**	–															
4.Hallucination	0.298**	0.546**	0.563**	–														
5.Agitation	0.109	0.668**	0.214*	0.261**	–													
6.Dysphoria	0.001	0.437**	0.191	0.318**	0.204*	–												
7.Anxiety	0.043	0.504**	0.249*	0.242*	0.218*	0.232*	–											
8.Euphoria	− 0.125	0.288**	0.123	0.124	0.083	0.015	0.074	–										
9.Apathy	0.098	0.484**	0.072	0.176	0.409**	0.299**	0.205*	− 0.086	–									
10.Disinhibition	− 0.033	0.478**	0.166	0.141	0.389**	− 0.027	0.106	0.128	0.075	–								
11.Irritability	− 0.046	0.624**	0.167	0.211*	0.359**	0.117	0.339**	0.173	0.173	0.475**	–							
12.Motor	− 0.015	0.506**	0.173	0.276**	0.296**	0.095	0.103	0.073	0.128	0.157	0.224*	–						
13.Sleep	− 0.086	0.528**	0.046	0.173	0.327**	0.227*	0.086	0.067	0.238*	0.141	0.196*	0.204*	–					
14.Eating	− 0.152	0.344**	− 0.123	− 0.135	0.159	0.046	0.062	0.204*	0.066	0.103	0.098	0.138	0.287**	–				
15.Age(c)	0.220*	− 0.029	− 0.103	0.071	− 0.034	− 0.031	0.009	0.081	− 0.055	− 0.111	− 0.023	− 0.135	0.168	0.018	–			
16.Sex(c)	0.020	− 0.047	− 0.316**	− 0.132	0.114	0.069	− 0.058	− 0.066	0.141	0.000	− 0.087	− 0.047	− 0.036	0.126	0.112	–		
17.PSS(c)	0.246*	0.311**	0.135	0.148	0.339**	0.082	0.145	0.027	0.256**	0.231*	0.253*	0.000	0.222*	− 0.008	− 0.000	− 0.102	–	
18.PHQ–9(c)	0.215*	0.254*	0.080	0.120	0.333**	0.248*	0.025	− 0.065	0.186	0.052	0.102	0.023	0.290**	0.084	0.052	0.009	0.490**	–
19.ZBI-22(c)	0.255*	0.369**	0.083	0.220*	0.460**	0.189	0.083	0.041	0.223*	0.285**	0.256**	0.047	0.284**	0.025	− 0.662	− 0.066	0.557**	0.510**

*AD* Alzheimer’s disease, *BPSD* behavioral and psychological symptoms of dementia, *NPI-*Q neuropsychiatric inventory questionnaires, *PSS* perceived stress scale, *PHQ-9* patient health questionnaire-9, *ZBI-22* 22-item Zarit Burden Interview.

*p-value < 0.05, **p-value < 0.01, c caregiver.

After adjustment for caregiver age, sex, relationship type, severity of AD, and other domains of BPSD, only agitation was strongly associated with PHQ-9 (coef 1.173, *p* = 0.021) and ZBI-22 (coef 4.792, *p* = 0.007); sleep disorders were associated with only PHQ-9 (coef 0.735, *p* = 0.049) (Table 5). The model fit statistics suggested that the models relatively fit the data for PSS model (adjusted R^2^ = 0.20, F = 2.63, *p*-value = 0.003, RMSE = 6.37), PHQ-9 model (adjusted R^2^ = 0.14, F = 2.06, *p*-value = 0.019, RMSE = 3.68), and ZBI-22 model (adjusted R^2^ = 0.21, F = 2.78, *p*-value = 0.001, RMSE = 12.68).

**Table 5:** Severity of BPSD and perceived stress, depression, and burden reported by caregivers (multivariable linear regression analysis).

	PSS		PHQ-9		ZBI-22	
	Coefficient* (SE)	*p*-value	Coefficient* (SE)	*p*-value	Coefficient* (SE)	*p*-value
Delusion	0.133 (0.742)	0.858	0.010 (0.449)	0.981	− 0.652 (1.573)	0.679
Hallucinations	0.179 (0.959)	0.853	− 0.163 (0.581)	0.780	0.769 (2.033)	0.706
Agitation	1.350 (0.823)	0.105	1.173 (0.499)	**0.021**	4.792 (1.738)	**0.007**
Dysphoria	− 0.725 (0.840)	0.391	0.728 (0.509)	0.157	1.645 (1.770)	0.356
Anxiety	0.259 (0.676)	0.703	− 0.329 (0.409)	0.424	− 0.758 (1.429)	0.597
Euphoria	− 1.339 (0.912)	0.884	− 0.350 (0.552)	0.528	0.427 (1.923)	0.825
Apathy	0.500 (0.669)	0.457	− 0.096 (0.405)	0.814	0.067 (1.415)	0.962
Disinhibition	0.799 (0.754)	0.293	− 0.129 (0.457)	0.778	2.115 (1.614)	0.194
Irritability	1.068 (0.724)	0.144	0.253 (0.439)	0.565	1.285 (1.540)	0.407
Aberrant motor behavior	− 0.726 (0.563)	0.201	− 0.317 (0.341)	0.355	− 1.280 (1.191)	0.286
Sleep disorders	1.009 (0.629)	0.112	0.735 (0.381)	**0.049**	1.805 (1.362)	0.189
Appetite/eating behavior	− 0.212 (0.591)	0.721	0.093 (0.358)	0.795	− 0.849 (1.283)	0.510

*AD* Alzheimer’s disease, *BPSD* behavioral and psychological symptoms of dementia, *PSS* perceived stress scale, *PHQ-9* patient health questionnaire-9, *SE* standard error, *ZBI-22* 22-item Zarit Burden Interview. Adjusted for caregiver age, sex, severity of AD, and other domains of BPSD.

Bold values indicate statistical significance (*p* ≤ 0.05).

*Unstandardized coefficients.

## Discussion

The findings of this study support the well substantiated high prevalence of BPSD in patients with AD. Our study found that most types of BPSD were not associated with disease severity except for hallucination. This finding adds to a growing literature on BPSD in patients with AD. Interestingly, the study showed that agitation was the only symptom that was strongly associated with both caregiver burden and depression.

The prevalence of BPSD in our study was as high as 99.0%. This high prevalence is non-surprising in light of previous research^28^. Our prevalence was similar to findings from many studies even though many of the studies focused on a variety of types of dementia. For example, a study in India found that 99.1% of patients with dementia in their cognitive unit had at least one BPSD^12^. A study in the memory clinic in a medical center in Taiwan reported an overall prevalence of BPSD of 87.6%, and the prevalence was higher in the more severe group^29^. Among those BPSD, apathy has been ranked highest which correlates with a prior study in AD^30^.

Current knowledge about the relationship between specific BPSD and severity of dementia are inconsistent^31–34^. In our study, hallucination was the only symptom among all 12 domains in which the severity of the symptom is related to the severity of AD. Even though, apathy (72.9%), sleep disorders (72.9%), and irritability (67.4%) were the most prevalent symptoms among patients with moderate-to-severe AD. This finding ties well with previous study wherein the frequency of hallucination increased across the various dementia stages^35^. It has been verified that hallucination is not common in AD, especially in the early stages, as in other types of dementia^36^. However, as the progression of the disease increasingly affects the brain pathology, this could be a common cause for hallucination in later stages of AD. The hallucinations in AD can be any types of hallucinations, the more common types being visual and auditory. A recent review reported that brain changes which could be associated with hallucination include occipital atrophy, and hypoperfusion in the left dorsolateral prefrontal, left medical temporal, and right parietal cortices^17^. In addition, the cholinergic deficiency commonly found in association with AD, would lead to hallucinations similar to those found in dementia of Lewy bodies^37^.

Contrary to the earlier findings that caregiver burden could be related to all type of BPSD^38^, we found caregiver burden was associated with only agitation after adjusting for caregiver age, sex, relationship type, severity of AD, and other domains of BPSD. Our results were in line with previous studies which found that agitation or aggressive behavior was the predictor of caregiver depressive symptoms^39,40^, even though more BPSD were found to be associated with the caregiver symptoms than the present study. Consistent with one study which reported that caregivers displayed higher burnout when facing agitation^41^, this association between patient agitation and caregiver outcome can be bidirectional. This symptom could be related to the inability to express needs verbally, for example, pain^13,42^ or unmet expectations^43^. Thus, the patients express those feelings with agitation which could impact the caregiver’s feelings resulting in stress and depression. On the other hand, this could be aggravated by acts of the caregiver when dealing with the patient. When they feel distress or burden, they may act or communicate inappropriately to the patient leading to inappropriate verbal, vocal, or motor behaviors as associated with agitation^44^. Apart from finding causes for agitation and providing appropriate treatment, non-pharmacological intervention such as music therapy, sensory intervention (therapeutic touch or massage), teaching communication, and coping skills for the caregiver could help reduce both agitation and caregiver distress^41^.

Sleep disorders or night-time behaviors, such as insomnia, wandering and actions inappropriate to a family situation tended to lead to depression in our study. The caregiver might perceive that they cannot rest in the night time in order to take care of their loved one. Sleep deprivation can cause depression via neurotransmitter changes^45–47^.

In contrast to the findings in our study, a variety of differing results have been published about BPSD and the well-being of caregivers. For example, research using a caregiver burden scale revealed that hallucinations, irritability, and depression were significant predictors of caregiver burden^48^. Another piece of research, which used the 10-item Chinese Center for Epidemiological Studies Depression Scale (CESD-10) for assessing caregiver depression and the Chinese Neuropsychiatric Inventory-Caregiver Distress Scale for assessing caregiver distress, found that all BPSD except for euphoria and night-time behavior are associated with caregiver depression and distress^49^. The difference between these results and ours may be due to the different measurement techniques used to measure depressive symptoms and burden in the caregiver^20^.

The strength of this study was the focus on AD not including the other types of dementia to ensure the focus was on the characteristic BPSD in AD. In addition, the caregivers who were interviewed in our study were the primary caregiver. Thus, the results could represent the effects on the main caregiver and be extrapolated in other similar situations. However, the study is not without its limitations. Due to the nature of its cross-sectional design, the temporal relationship could not be determined. Also the study was conducted in a single center; therefore, the results could differ in another context and need to be reviewed as regards reproducibility in other settings. The limitation about our small sample size should be noted. Further studies with larger samples are needed to confirm the result from our study.

In conclusion, this study showed a high prevalence of BPSD in AD patients, and the symptoms can start early in the disease process. The BPSD, especially agitation and sleep disorders, can give rise to difficulties for both patients and their caregivers which highlights the benefits of treating this specific behavioral symptom for patients and caregivers. Our findings provide further support for routine screening and treating BPSD in AD patients.

## Methods

### Study design

We conducted a cross-sectional study involving the interviewing of primary caregivers of AD patients who were treated in neurology clinic at Chiang Mai University Hospital, a tertiary care hospital in Northern Thailand. This study was approved by the research ethics committee of the Faculty of Medicine, Chiang Mai University (346/2560). All methods were performed in accordance with the relevant guidelines and regulations. All participants gave written informed consent before completing the questionnaires.

### Data collection

Caregivers aged 18 years-old or more, who had been providing care for at least 1 month, were recruited. Exclusion criteria was inability to communicate. The caregivers were recruited by convenience sampling. Caregivers were interviewed to evaluate patient status and their own personal data. The severity of the disease of the patients and their level of function were evaluated through the perceptions of the caregivers by using FAST, Barthel index, NPI-Q. Caregiver outcomes included stress, burden, and depression which were assessed using PSS, ZBI-22, and PHQ-9.

### Measures

#### Functional assessment staging test (FAST)

This scale was used to determine the stage of AD. It consists of seven stages (1–7 stages). The higher the stage, the more severe the dementia. The patients were categorized into two group, mild dementia (stage 2–3) and moderate-to-severe dementia (stage 4–7)^22^.

#### Neuropsychiatric inventory questionnaires (NPI-Q)

The NPI-Q is a questionnaire which is used to assess 12 domains of BPSD during the past month by asking informants if the symptom is present and the severity of the symptoms, ranging from mild to severe^24^. The 12 domains include delusion, hallucinations, agitation, dysphoria, anxiety, euphoria, apathy, disinhibition, irritability, aberrant motor behavior, sleep disorders, and eating behavior.

#### Barthel index

The Barthel index was used to assess activities daily living (ADL) of the patient. It consists of 10 basic domains^23^. The score has a range of 0–20, higher scores indicating a better daily function.

#### Perceived stress scale (PSS)

The PSS is a 10-item self-reporting questionnaire measuring the perceived level of stress in the individual^25^. The score has a range of 0–40, higher scores indicating a greater level of perceived stress.

#### Patient health questionnaire-9 (PHQ-9)

The PHQ-9 is a 9-item self-reporting questionnaire measuring depressive symptoms over the past 2 weeks^26^. The score has a range of 0–27, higher scores indicating a greater depressive symptoms.

#### Zarit burden interview (ZBI-22)

The ZBI-22 is a 22-item caregiver-reported questionnaire measuring caregiver burden specifically the perception of the caregiver pertinent to the emotional, social, and financial consequences of providing care for someone^27^. The score has a range of 0–88, higher scores indicating a greater caregiving burden.

The summary of all assessments measures is described in Supplementary Table S1.

### Statistical analysis

All analyses were performed using STATA SE 15.1 (Stata Corp LCC, College Station, TX, USA)^50^. The descriptive data are displayed in frequencies, percentages, mean, and standard deviation (SD). The association between severity of BPSD and severity of AD was determined using a Chi-square test and Fisher’s exact test where appropriate. Assessment of the association between caregiver outcome and severity of BPSD was made using Pearson’s correlation. Multivariable linear regression analysis models were then used to determine the association adjusted for other domains of BPSD of the patients. Variables including age, sex, and relationship type were priori confounders because they were known risk factors for the outcome of interest^51–54^ and always included in the multivariable model. Correlation analyses were conducted to assess the associations between all variables to be included in the analysis model. The unstandardized coefficients were used. *p*-values ≤ 0.05 were considered statistically significant.

## Supplementary Information

Supplementary Information.

## Data Availability

The data that support the findings of this study are available from the corresponding author upon reasonable request.

## References

[1] World Health Organization. *Global Action Plan on the Public Health Response to Dementia 2017–2025*. (Geneva, 2017).

[2] Mayeux, R. & Stern, Y. Epidemiology of Alzheimer disease. *Cold Spring Harb. Perspect. Med.* **2**, a006239. 10.1101/cshperspect.a006239 (2012). 22908189 10.1101/cshperspect.a006239 PMC3405821

[3] Brickman, A. M. *et al.* Longitudinal assessment of patient dependence in Alzheimer disease. *Arch. Neurol.* **59**, 1304–1308. 10.1001/archneur.59.8.1304 (2002). 12164728 10.1001/archneur.59.8.1304

[4] McLaughlin, T. *et al.* Dependence as a unifying construct in defining Alzheimer’s disease severity. *Alzheimers Dement.* **6**, 482–493. 10.1016/j.jalz.2009.09.004 (2010). 21044778 10.1016/j.jalz.2009.09.004 PMC3884683

[5] Chiu, M. J., Chen, T. F., Yip, P. K., Hua, M. S. & Tang, L. Y. Behavioral and psychologic symptoms in different types of dementia. *J.Formos. Med. Assoc.* **105**, 556–562. 10.1016/s0929-6646(09)60150-9 (2006). 16877235 10.1016/S0929-6646(09)60150-9

[6] Cerejeira, J., Lagarto, L. & Mukaetova-Ladinska, E. B. Behavioral and psychological symptoms of dementia. *Front. Neurol.* **3**, 73. 10.3389/fneur.2012.00073 (2012). 22586419 10.3389/fneur.2012.00073 PMC3345875

[7] Alzheimer’s Disease International & Dementia Australia. *Dementia in the Asia Pacific region*. 60 (London, 2014).

[8] Chuakhamfoo, N. N., Phanthunane, P., Chansirikarn, S. & Pannarunothai, S. Health and long-term care of the elderly with dementia in rural Thailand: A cross-sectional survey through their caregivers. *BMJ Open* **10**, e032637. 10.1136/bmjopen-2019-032637 (2020). 32209620 10.1136/bmjopen-2019-032637 PMC7202699

[9] Shim, Y. S. *et al.* Caregiving, care burden and awareness of caregivers and patients with dementia in Asian locations: A secondary analysis. *BMC Geriatr.* **21**, 230. 10.1186/s12877-021-02178-x (2021). 33827446 10.1186/s12877-021-02178-x PMC8028783

[10] Muangpaisan, W. *et al.* Caregiver burden and needs of dementia caregivers in Thailand: A cross-sectional study. *J. Med. Assoc. Thai.* **93**, 601–607 (2010). 20524447

[11] Lamliangpon, P. Anxiety-depression and quality of life in dementia caregivers. *J. Ment Health Thail.* **23**, 113–124 (2015).

[12] Mukherjee, A. *et al.* Behavioural and psychological symptoms of dementia: Correlates and impact on caregiver distress. *Dement. Geriatr. Cognit. Disord. Extra* **7**, 354–365. 10.1159/000481568 (2017). 10.1159/000481568 PMC5731149 29282408

[13] Arthur, P. B., Gitlin, L. N., Kairalla, J. A. & Mann, W. C. Relationship between the number of behavioral symptoms in dementia and caregiver distress: What is the tipping point?. *Int. Psychogeriatr.* **30**, 1099–1107. 10.1017/s104161021700237x (2018). 29143722 10.1017/S104161021700237X PMC7103581

[14] Miyamoto, Y., Tachimori, H. & Ito, H. Formal caregiver burden in dementia: Impact of behavioral and psychological symptoms of dementia and activities of daily living. *Geriatr. Nurs.* **31**, 246–253. 10.1016/j.gerinurse.2010.01.002 (2010). 20682402 10.1016/j.gerinurse.2010.01.002

[15] Hurt, C. *et al.* Patient and caregiver perspectives of quality of life in dementia. An investigation of the relationship to behavioural and psychological symptoms in dementia. *Dement. Geriatr. Cogn. Disord.* **26**, 138–146. 10.1159/000149584 (2008). 18679028 10.1159/000149584

[16] Tan, L. L., Wong, H. B. & Allen, H. The impact of neuropsychiatric symptoms of dementia on distress in family and professional caregivers in Singapore. *Int. Psychogeriatr.* **17**, 253–263. 10.1017/s1041610205001523 (2005). 16050434 10.1017/s1041610205001523

[17] Gottesman, R. T. & Stern, Y. Behavioral and psychiatric symptoms of dementia and rate of decline in Alzheimer’s disease. *Front. Pharmacol.* **10**, 1062. 10.3389/fphar.2019.01062 (2019). 31616296 10.3389/fphar.2019.01062 PMC6768941

[18] Pinyopornpanish, M. *et al.* Perceived stress and depressive symptoms not neuropsychiatric symptoms predict caregiver burden in Alzheimer’s disease: A cross-sectional study. *BMC Geriatr.* **21**, 180. 10.1186/s12877-021-02136-7 (2021). 33711938 10.1186/s12877-021-02136-7 PMC7953798

[19] Ornstein, K. & Gaugler, J. E. The problem with “problem behaviors”: A systematic review of the association between individual patient behavioral and psychological symptoms and caregiver depression and burden within the dementia patient-caregiver dyad. *Int. Psychogeriatr.* **24**, 1536–1552. 10.1017/s1041610212000737 (2012). 22612881 10.1017/S1041610212000737 PMC5769475

[20] Feast, A., Moniz-Cook, E., Stoner, C., Charlesworth, G. & Orrell, M. A systematic review of the relationship between behavioral and psychological symptoms (BPSD) and caregiver well-being. *Int. Psychogeriatr.* **28**, 1761–1774. 10.1017/s1041610216000922 (2016). 27345942 10.1017/S1041610216000922

[21] Cloak, N. & Khalili, Y. A. *Behavioral and Psychological Symptoms in Dementia (BPSD)*, https://www.ncbi.nlm.nih.gov/books/NBK551552/ (2020).

[22] Sclan, S. G. & Reisberg, B. Functional assessment staging (FAST) in Alzheimer’s disease: Reliability, validity, and ordinality. *Int. Psychogeriatr.* **4**(Suppl 1), 55–69. 10.1017/s1041610292001157 (1992). 1504288 10.1017/s1041610292001157

[23] Mahoney, F. I. & Barthel, D. W. Functional evaluation: The barthel index. *Md State Med. J.* **14**, 61–65 (1965). 14258950

[24] Kaufer, D. I. *et al.* Assessing the impact of neuropsychiatric symptoms in Alzheimer’s disease: The neuropsychiatric inventory caregiver distress scale. *J. Am. Geriatr. Soc.* **46**, 210–215. 10.1111/j.1532-5415.1998.tb02542.x (1998). 9475452 10.1111/j.1532-5415.1998.tb02542.x

[25] Cohen, S., Kamarck, T. & Mermelstein, R. A global measure of perceived stress. *J. Health Soc. Behav.* **24**, 385–396 (1983). 6668417

[26] Kroenke, K., Spitzer, R. L. & Williams, J. B. The PHQ-9: Validity of a brief depression severity measure. *J. Gen. Intern. Med.* **16**, 606–613. 10.1046/j.1525-1497.2001.016009606.x (2001). 11556941 10.1046/j.1525-1497.2001.016009606.x PMC1495268

[27] Zarit, S. H., Reever, K. E. & Bach-Peterson, J. Relatives of the impaired elderly: Correlates of feelings of burden. *Gerontologist* **20**, 649–655. 10.1093/geront/20.6.649 (1980). 7203086 10.1093/geront/20.6.649

[28] Deardorff, W. J. & Grossberg, G. T. Behavioral and psychological symptoms in Alzheimer’s dementia and vascular dementia. *Handb. Clin. Neurol.* **165**, 5–32. 10.1016/b978-0-444-64012-3.00002-2 (2019). 31727229 10.1016/B978-0-444-64012-3.00002-2

[29] Huang, S.-S., Wang, W.-F. & Liao, Y.-C. Severity and prevalence of behavioral and psychological symptoms among patients of different dementia stages in Taiwan. *Arc. Clin. Psychiatr* **44**, 89–93. 10.1590/0101-60830000000127 (2017).

[30] Kazui, H. *et al.* Differences of behavioral and psychological symptoms of dementia in disease severity in four major dementias. *PLoS ONE* **11**, e0161092. 10.1371/journal.pone.0161092 (2016). 27536962 10.1371/journal.pone.0161092 PMC4990196

[31] Lopez, O. L. *et al.* Psychiatric symptoms vary with the severity of dementia in probable Alzheimer’s disease. *J. Neuropsychiatr. Clin. Neurosci.* **15**, 346–353. 10.1176/jnp.15.3.346 (2003). 10.1176/jnp.15.3.346 12928511

[32] Canevelli, M. *et al.* Impact of behavioral subsyndromes on cognitive decline in Alzheimer’s disease: Data from the ICTUS study. *J. Neurol.* **260**, 1859–1865. 10.1007/s00415-013-6893-3 (2013). 23504051 10.1007/s00415-013-6893-3

[33] Tanaka, H. *et al.* Relationship between dementia severity and behavioural and psychological symptoms in early-onset Alzheimer’s disease. *Psychogeriatrics* **15**, 242–247. 10.1111/psyg.12108 (2015). 25737233 10.1111/psyg.12108

[34] Hashimoto, M. *et al.* Relationship between dementia severity and behavioral and psychological symptoms of dementia in dementia with lewy bodies and Alzheimer’s disease patients. *Dement. Geriatr. Cogn. Dis. Extra* **5**, 244–252. 10.1159/000381800 (2015). 26195980 10.1159/000381800 PMC4483492

[35] Piccininni, M., Di Carlo, A., Baldereschi, M., Zaccara, G. & Inzitari, D. Behavioral and psychological symptoms in Alzheimer’s disease: Frequency and relationship with duration and severity of the disease. *Dement. Geriatr. Cogn. Disord.* **19**, 276–281. 10.1159/000084552 (2005). 15775717 10.1159/000084552

[36] Linszen, M. M. J. *et al.* Understanding hallucinations in probable Alzheimer’s disease: Very low prevalence rates in a tertiary memory clinic. *Alzheimers Dement. (Amst)* **10**, 358–362. 10.1016/j.dadm.2018.03.005 (2018). 30014034 10.1016/j.dadm.2018.03.005 PMC6019263

[37] Dauwan, M. *et al.* EEG-based neurophysiological indicators of hallucinations in Alzheimer’s disease: Comparison with dementia with Lewy bodies. *Neurobiol. Aging* **67**, 75–83. 10.1016/j.neurobiolaging.2018.03.013 (2018). 29653315 10.1016/j.neurobiolaging.2018.03.013

[38] Kim, B., Noh, G. O. & Kim, K. Behavioural and psychological symptoms of dementia in patients with Alzheimer’s disease and family caregiver burden: A path analysis. *BMC Geriatr.* **21**, 160–160. 10.1186/s12877-021-02109-w (2021). 33663416 10.1186/s12877-021-02109-w PMC7934246

[39] Fauth, E. B. & Gibbons, A. Which behavioral and psychological symptoms of dementia are the most problematic? Variability by prevalence, intensity, distress ratings, and associations with caregiver depressive symptoms. *Int. J. Geriatr. Psychiatry* **29**, 263–271. 10.1002/gps.4002 (2014). 23846797 10.1002/gps.4002

[40] Covinsky, K. E. *et al.* Patient and caregiver characteristics associated with depression in caregivers of patients with dementia. *J. Gen. Intern. Med.* **18**, 1006–1014. 10.1111/j.1525-1497.2003.30103.x (2003). 14687259 10.1111/j.1525-1497.2003.30103.x PMC1494966

[41] Hiyoshi-Taniguchi, K., Becker, C. B. & Kinoshita, A. What behavioral and psychological symptoms of dementia affect caregiver burnout?. *Clin. Gerontol.* **41**, 249–254. 10.1080/07317115.2017.1398797 (2018). 29252121 10.1080/07317115.2017.1398797

[42] Jenny Wei, Y.-J. *et al.* Uncontrolled pain and risk for depression and behavioral symptoms in residents with dementia. *J. Am. Med. Dir. Assoc.* **22**, 2079-2086.e2075. 10.1016/j.jamda.2021.05.010 (2021). 34089652 10.1016/j.jamda.2021.05.010 PMC8478709

[43] Kales, H. C., Gitlin, L. N. & Lyketsos, C. G. Assessment and management of behavioral and psychological symptoms of dementia. *BMJ Open*10.1136/bmj.h369 (2015). 10.1136/bmj.h369 PMC4707529 25731881

[44] Livingston, G. *et al.* Non-pharmacological interventions for agitation in dementia: Systematic review of randomised controlled trials. *Br. J. Psychiatry* **205**, 436–442. 10.1192/bjp.bp.113.141119 (2014). 25452601 10.1192/bjp.bp.113.141119

[45] Franzen, P. L. & Buysse, D. J. Sleep disturbances and depression: Risk relationships for subsequent depression and therapeutic implications. *Dialog. Clin. Neurosci.* **10**, 473–481. 10.31887/DCNS.2008.10.4/plfranzen (2008). 10.31887/DCNS.2008.10.4/plfranzen PMC3108260 19170404

[46] Roberts, R. E. & Duong, H. T. The prospective association between sleep deprivation and depression among adolescents. *Sleep* **37**, 239–244. 10.5665/sleep.3388 (2014). 24497652 10.5665/sleep.3388 PMC3900610

[47] Novati, A. *et al.* Chronically restricted sleep leads to depression-like changes in neurotransmitter receptor sensitivity and neuroendocrine stress reactivity in rats. *Sleep* **31**, 1579–1585. 10.1093/sleep/31.11.1579 (2008). 19014078 10.1093/sleep/31.11.1579 PMC2579986

[48] Torrisi, M. *et al.* Neuropsychiatric symptoms in dementia may predict caregiver burden: A Sicilian exploratory study. *Psychogeriatrics* **17**, 103–107. 10.1111/psyg.12197 (2017). 27411501 10.1111/psyg.12197

[49] Chiu, Y.-C. *et al.* Family caregivers’ sleep disturbance and its associations with multilevel stressors when caring for patients with dementia. *Aging Ment. Health* **18**, 92–101. 10.1080/13607863.2013.837141 (2014). 24053456 10.1080/13607863.2013.837141

[50] Stata Statistical Software. *Release 15* (StataCorp LLC, 2017).

[51] Jorm, A. F. Sex and age differences in depression: A quantitative synthesis of published research. *Aust. N. Z. J. Psychiatry* **21**, 46–53. 10.3109/00048678709160898 (1987). 3497630 10.3109/00048678709160898

[52] Xiong, C. *et al.* Sex and gender differences in caregiving burden experienced by family caregivers of persons with dementia: A systematic review. *PLoS ONE* **15**, e0231848. 10.1371/journal.pone.0231848 (2020). 32310969 10.1371/journal.pone.0231848 PMC7170244

[53] Groenwold, R. H., Klungel, O. H., Grobbee, D. E. & Hoes, A. W. Selection of confounding variables should not be based on observed associations with exposure. *Eur. J. Epidemiol.* **26**, 589–593. 10.1007/s10654-011-9606-1 (2011). 21796419 10.1007/s10654-011-9606-1 PMC3168755

[54] Connors, M. H. *et al.* Dementia and caregiver burden: A 3-year longitudinal study. *Int. J. Geriatr. Psychiatry* **35**, 250–258. 10.1002/gps.5244 (2020). 31821606 10.1002/gps.5244

