# Author Correction: Impact of behavioral and psychological symptoms of Alzheimer’s disease on caregiver outcomes

**DOI:** 10.1038/s41598-025-02780-8

**Published:** 2025-06-06

**Authors:** Kanokporn Pinyopornpanish, Atiwat Soontornpun, Tinakon Wongpakaran, Nahathai Wongpakaran, Surat Tanprawate, Kanokwan Pinyopornpanish, Angkana Nadsasarn, Manee Pinyopornpanish

**Affiliations:** 1 Department of Family Medicine, Faculty of Medicine, Chiang Mai University, Chiang Mai, Thailand; 2 Division of Neurology, Department of Internal Medicine, Faculty of Medicine, Chiang Mai University, 110 Inthawarorot Rd., Sriphum, Muang, Chiang Mai, 50200 Thailand; 3 Northern Neuroscience Center, Faculty of Medicine, Chiang Mai University, Chiang Mai, Thailand; 4 Department of Psychiatry, Faculty of Medicine, Chiang Mai University, Chiang Mai, Thailand

Correction to: *Scientific Reports* 10.1038/s41598-022-18470-8, published online 19 August 2022

The original version of this Article contained an error in the Methods section, under the subheading ‘Study design’,

“This study was approved by the research ethics committee of the Faculty of Medicine, Chiang Mai University (PSY-2560-05,110).”

now reads:

“This study was approved by the research ethics committee of the Faculty of Medicine, Chiang Mai University (346/2560).”

The original Article has been corrected.

