# Supplementary material for: Impact of behavioral and psychological symptoms of Alzheimer’s disease on caregiver outcomes
Source: Sci Rep. 2022 Aug 19;12:14138. doi: 10.1038/s41598-022-18470-8 (PMC9391353; doi:10.1038/s41598-022-18470-8)
Supplement: Supplementary file 1 — Supplementary Information. [file 41598_2022_18470_MOESM1_ESM.docx]

**Table S1** Summary of assessment measures

| Measure | Purpose | Description |
| --- | --- | --- |
| Functional Assessment Staging Test (FAST) | To stage a patient's level of disability by using information obtained from knowledgeable informant. | It consists of 7 major stages of functioning (1-7), with stage 1 representing no functional decline and stage 7 representing severe dementia . |
| Neuropsychiatric Inventory Questionnaires (NPI-Q) | Measure presence, severity of 12 domains of BPSD by asking informants. | For each of 12 domains, caregivers note "yes or no" and rated within a domain in terms of severity (1=mild, 2=moderate, and 3=severe), thus yielding a score range from 0-36. |
| Barthel Index | Assess performance in activities of daily living (ADL). Ten domains describing ADL and mobility are scored. | Scores range from 0-20, where higher number being reflection of greater ability to function independently. |
| Perceived Stress Scale (PSS) | Measure perceived level of stress with 10 items. | Scores range from 0-40, where high scores imply greater level of perceived stress |
| Patient Health Questionnaire-9 (PHQ-9) | Measure depressive symptoms in caregivers with 9 items. | Scores range from 0-27, where high scores suggest greater depressive symptoms. |
| Zarit Burden Interview (ZBI-22) | Measure caregiver burden when caring for cognitively impaired adults with 22 items | Scores range from 0-88, where high scores imply greater caregiver burden. |
